# Correlation between quantification of myocardial area at risk and ischemic burden at cardiac computed tomography

**DOI:** 10.1016/j.ejro.2022.100417

**Published:** 2022-03-31

**Authors:** F.Y. van Driest, C.M. Bijns, R.J. van der Geest, A. Broersen, J. Dijkstra, J.W. Jukema, A.J.H.A. Scholte

**Affiliations:** a Department of Cardiology, Leiden Heart-Lung Center, Leiden University Medical Center, Leiden, The Netherlands; b Department of Radiology, Division of Image Processing, Leiden University Medical Center, Leiden, The Netherlands

**Keywords:** AUC;, Area under the curve, CAD;, Coronary artery disease, CCTA;, Coronary computed tomography angiography, CTP;, Computed tomography perfusion, CX;, Circumflex artery, ECG;, Electrocardiogram, FFR;, Fractional flow reserve, LAD;, Left anterior descending artery, LV;, Left ventricle, MBF;, Myocardial blood flow, MRI;, Magnetic resonance imaging, RCA;, Right coronary artery, SPECT;, Single photon emission computed tomography, VTK;, Visualization toolkit, Coronary computed tomography angiography, Myocardial computed tomography perfusion, Algorithms, Myocardial area at risk, Myocardial ischemia

## Abstract

**Purpose:**

This study aims to investigate the correlation between myocardial area at risk at coronary computed tomography angiography (CCTA) and the ischemic burden derived from myocardial computed tomography perfusion (CTP) by using the 17-segment model.

**Methods:**

Forty-two patients with chest pain complaints who underwent a combined CCTA and CTP protocol were identified. Patients with reversible ischemia at CTP and at least one stenosis of ≥ 50% at CCTA were selected. Myocardial area at risk was calculated using a Voronoi-based segmentation algorithm at CCTA and was defined as the sum of all territories related to a ≥ 50% stenosis as a percentage of the total left ventricular (LV) mass. The latter was calculated using LV contours which were automatically drawn using a machine learning algorithm. Subsequently, the ischemic burden was defined as the number of segments demonstrating relative hypoperfusion as a percentage of the total amount of segments (=17). Finally, correlations were tested between the myocardial area at risk and the ischemic burden using Pearson’s correlation coefficient.

**Results:**

A total of 77 coronary lesions were assessed. Average myocardial area at risk and ischemic burden for all lesions was 59% and 23%, respectively. Correlations for ≥ 50% and ≥ 70% stenosis based myocardial area at risk compared to ischemic burden were moderate (r = 0.564; p < 0.01) and good (r = 0.708; p < 0.01), respectively.

**Conclusion:**

The relation between myocardial area at risk as calculated by using a Voronoi-based algorithm at CCTA and ischemic burden as assessed by CTP is dependent on stenosis severity.

## 1 Introduction

Coronary computed tomography angiography (CCTA) is widely used to diagnose coronary artery disease (CAD) and determine stenosis severity [1]. However, the assessment of ischemic myocardium is also of prognostic importance and plays a vital role in the decision to revascularize patients which depends on the extent of the relative hypoperfused (ischemic) myocardium, relative to the subtended myocardial mass distal of the coronary stenosis [2]. A key advantage of combining CCTA and adenosine stress CT myocardial perfusion (CTP) is that it allows for both the assessment of coronary artery stenosis as well as myocardial ischemia [2]. Also, CTP has a substantially shorter exam time as compared to cardiac magnetic resonance (CMR) and myocardial perfusion imaging (MPI). Furthermore, CTP may be especially beneficial in patients with contraindications for CMR [3], [4]. However, it must be noted that a major disadvantage of CTP is the relatively high radiation dose exposure. Still, this is gradually improving thanks to technological advancement [4].

The Voronoi decomposition encompasses a mathematical algorithm that divides a three-dimensional space or two-dimensional area between predetermined points based on the shortest distance to those points. This algorithm can be used to partition the myocardium according to which blood vessel is closest [5], [6]. By using a Voronoi decomposition algorithm on myocardial tissue one can take into account the many variations that exist in coronary anatomy. This is a major advantage of the aforementioned method over the standard 17 segment model in which the segments correspond to a fixed location and do not change according to differences in coronary anatomy [7]. The importance of using a different approach for the assessment of the coronary distribution was demonstrated in a study by Ortiz­Perez et al. in which in patients who underwent CMR 23% of the hyper enhanced segments were discordant with the empirically assigned coronary distribution according to the standard 17­segment model. A Voronoi based segmentation algorithm can overcome this problem as its output is dependent on patient specific coronary anatomy [6], [8].

Artificial intelligence (AI) is rapidly evolving in the work field of cardiovascular imaging and can greatly lessen the time needed for image processing, Machine learning which is a subclass of AI allows for the creation of a model based on historical data. As such, machine learning has been widely used for automatic left ventricle (LV) segmentation greatly speeding up the process of LV contour placement [9], [10].

The aim of this study was to assess whether the subtended myocardial mass as calculated by using the Voronoi-based segmentation method correlated to myocardial ischemia at CTP. As such, CCTA may not only be used to assess the degree of a coronary stenosis, but also for the quantification of the subtended myocardial mass which may predict the ischemic burden without the need for a stress test.

## 2 Materials and methods

### 2.1 Patients

**Fig. 1:**
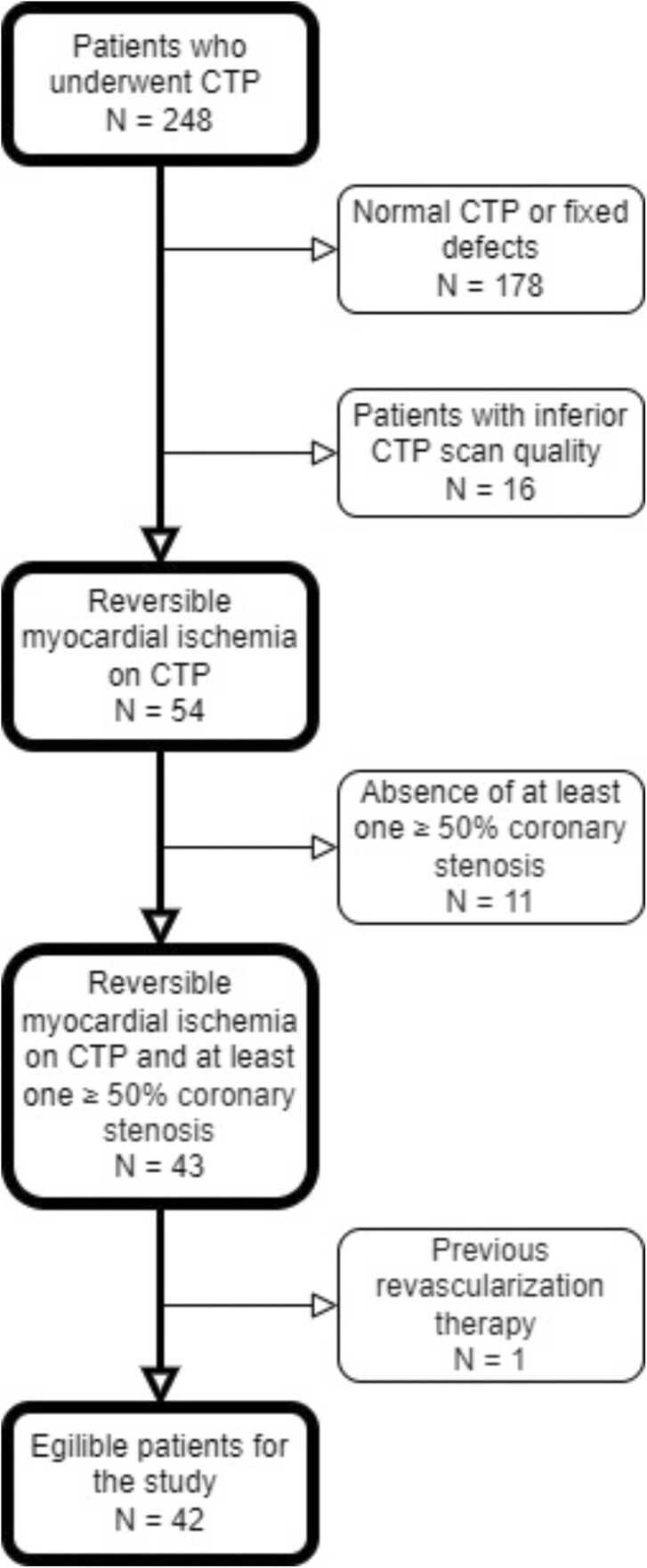
Flowchart depicting the selection process of patients. CTP scans with “poor” or “fair” scan quality were deemed inferior.

248 patients referred for a combined CCTA and CTP protocol due to chest pain complaints were identified. Patients with normal CTP images or fixed perfusion defects (N═178), absence of at least one ≥ 50% coronary stenosis (N = 11), inferior CTP scan quality (N = 16) and prior coronary revascularization (N = 1) were excluded [11]. We selected a total of 42 patients for the current analysis. A detailed flowchart of the patient selection is depicted in Fig. 1. CTP scan quality classified as either “poor” or “ fair” was deemed inferior. All data were retrospectively analyzed. The local ethics committee of the Leiden University Medical Center approved this retrospective analysis of clinical data and the need for informed consent was waived.

### 2.2 Data acquisition

Using a 320-row volumetric scanner (Aquilion ONE, Canon Medical Systems and Aquilion ONE Genesis Edition, Canon Medical Systems, Otawara, Japan) CCTA and static adenosine stress CTP were acquired on the same day. Patients were advised not to consume caffeine products 24 h before examination. One hour prior to CCTA blood pressure and heart rate were monitored. Patients with a heart rate exceeding 60 beats per minutes (bpm) were given metoprolol, 25 mg up to 150 mg orally, unless contraindications were present. Additionally, metoprolol could be injected intravenously if the heart rate remained above 60 bpm.

Sublingual administration of nitroglycerin (0.4 mg) was done prior to CCTA. Scanner settings for CCTA were as follows: A detector collimation of 320 × 0.5 mm, a 275 ms gantry rotation time and temporal resolution of 137 ms for the Aquilion ONE Genesis Edition and a detector collimation of 320 × 0.5 mm, 350 ms gantry rotation time and temporal resolution of 175 ms for the Aquilion ONE. Tube current was 140–580 mA and a peak tube voltage 100–135 kV. The antecubital vein was used for administration of 50–90 mL of contrast agent (Iomeron 400, Bracco, Milan, Italy) followed by a 1:1 mixture of 20 mL contrast and saline and finally 25 mL of saline. Tube current, peak tube voltage and the amount of administered contrast agent varied due to variations in patient size [12]. Using prospective electrocardiogram (ECG) triggering 70–80% of the RR interval was scanned. In patients with a heart rate exceeding 65 bpm 30–80% of the RR-interval was scanned. When a threshold of 300 Hounsfield units (HU) was reached in the descending aorta CCTA was performed the next beat.

CTP was only performed if there was suspicion of a significant stenosis (≥ 50%) at CCTA. To achieve adequate myocardial contrast wash-out the minimum scan-interval was 20 min between CCTA and CTP. ECG and blood pressure were continuously monitored following continuous adenosine infusion (0.14 mg/kg/min) after which a contrast agent was administered. CTP images were acquired when a threshold of 300 HU was reached in the descending aorta scanning 80–99% of the RR interval. Tube settings, injection protocol and contrast agent were all similar to the CCTA acquisition.

### 2.3 Image analysis

Images were transferred to a workstation and analyzed using dedicated post-processing software (Vitrea FX 7.12; Vital Images, Minnetonka, Minnesota). All CCTA and CTP images were analysed by trained cardiologists with at least 10 years of experience. In accordance with SCCT guidelines, stenosis severity per segment was semi quantitatively assessed using visual analysis as: 50–69% (moderate), 70–99% (severe), and 100% (occluded) [13]. In case multiple stenoses were observed in the same segment and vessel, the most proximal stenosis was labelled as the culprit stenosis.

CTP images were analysed by reconstructing cardiac phases for every 2% of the scanned interval. Subsequently, analysis was performed on the phase with the best scan quality using short-axis reformatted images and a slice thickness of 4 mm using a narrow window width and level setting (W300/L150) and utilizing the standard 17 segment myocardial model for scoring [14]. If one or more segments demonstrated signs of relative hypoperfusion the CTP was considered abnormal [11]. The number of segments with relative hypoperfusion relative to the total of 17 segments was defined as the ischemic burden and calculated using the following formula:

$$\mathrm{Ischemic}\;\mathrm{burden}=\frac{\mathrm{number}\;\mathrm{of}\;\mathrm{segments}\;\mathrm{with}\;\mathrm{relative}\;\mathrm{hypoperfusion}}{17}*100$$

### 2.4 Image processing

**Fig. 2:**
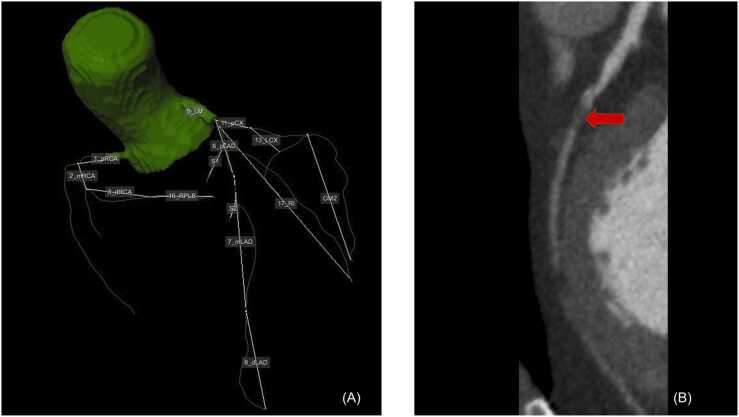
The complete coronary tree was automatically extracted from the CCTA (Panel A.). The proximal part of the lesion in the proximal LAD as marked by the red arrow (Panel B) is used as the starting point for calculating the subtended mass. (For interpretation of the references to color in this figure legend, the reader is referred to the web version of this article.)

**Fig. 3:**
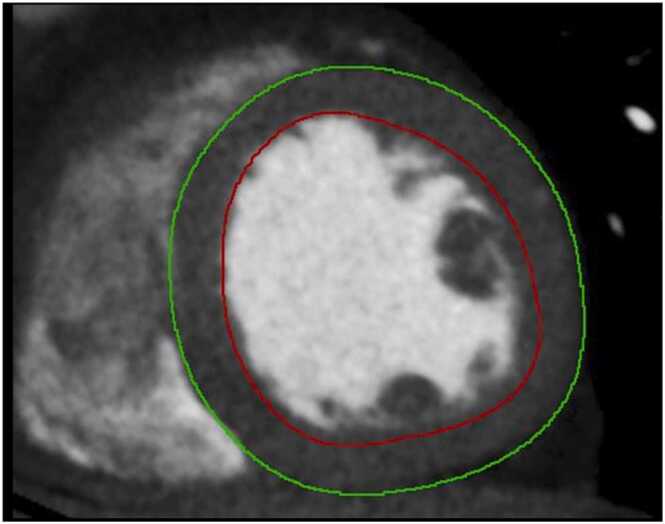
Epicardial contours (green line) and endocardial contours (red line) were automatically drawn using a machine learning model. (For interpretation of the references to color in this figure legend, the reader is referred to the web version of this article.)

Before executing the Voronoi-based segmentation algorithm the complete coronary artery tree was automatically extracted from the CCTA (Fig. 2A) and the relevant lesions were manually defined using dedicated software (Fig. 2B) (QAngio CT Research Edition v3.1.5.1 Medis Medical Imaging, Leiden, The Netherlands). Hereafter, the CCTA images were automatically reformatted into a short-axis orientation covering the complete left ventricle with an inter-slice spacing of 4 mm. Subsequently, left ventricular epicardial and endocardial contours were automatically drawn in the CCTA (Fig. 3). Both tasks were done semi automatically using in house developed MASS software (Leiden University Medical Center) by using a machine learning model, manual corrections were made if needed. This model was trained using a different dataset of 50 randomly selected CCTA’s in which reformatting of the short axis and drawing of the LV epicardial and endocardial contours was done manually. Subsequently we used dedicated open-source software (TensorFlow v2.6 software available from www.tensorflow.org) to train a neural network. Executing the machine learning model took approximately 1 min and 20 s per CCTA.

To assess the feasibility of the machine learning model as compared to manual measurements one observer (F.Y. with 3 years of experience in cardiovascular imaging analysis) randomly selected a sample of 10 cases in which manual reformatting of the short axis and manual drawing of the left ventricular epicardial and endocardial contours was performed. Correlations were subsequently tested between manual and automatic measurements concerning the left ventricular mass which is derived from the epicardial and endocardial contours. Statistical analysis of these correlations was done using Pearson’s correlation coefficient using SPSS software (version 25, SPSS IBM Corp, Armonk, New York).

### 2.5 Voronoi-based segmentation

**Fig. 4:**
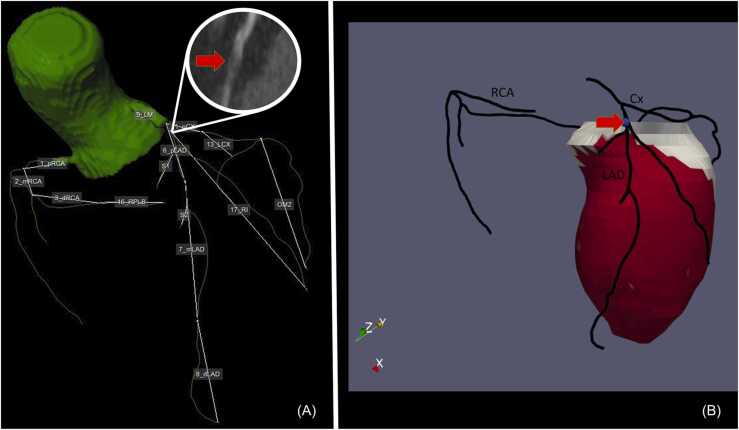
Using the previously defined lesion in the proximal LAD (Panel A) and executing the Voronoi-based algorithm the subtended mass can be computed and visualized in 3D (Panel B).

In order to calculate the subtended mass a Voronoi-based segmentation algorithm was used on the CCTA by using in-house developed MASS software (Leiden University Medical Center). By using this algorithm it is possible to find the nearest location of the extracted coronary artery tree for every voxel within the left ventricular myocardium [5], [6]. Subsequently, results of the image segmentation were exported as 3D objects in the visualization toolkit (VTK) format for further analysis and visualization (Fig. 4). Executing the Voronoi-based segmentation algorithm took approximately 1 min per lesion.

Finally, the subtended mass was calculated for both ≥ 50% and ≥ 70% stenosis as a percentage of the total LV mass and defined as the myocardial area at risk using the following formula:

$$\mathrm{myocardial}\;\mathrm{area}\;\mathrm{at}\;\mathrm{risk}=\frac{\mathrm{Subtended}\;\mathrm{mass}}{\mathrm{LV}\;\mathrm{mass}}*100$$

### 2.6 Statistical analysis

Correlations between the ischemic burden and myocardial area at risk as well as correlations between manual and machine learning based LV contours were calculated using Pearson’s correlation coefficient. All analysis were performed using SPSS software (version 25, SPSS IBM Corp, Armonk, New York).

## 3 Results

**Fig. 5:**
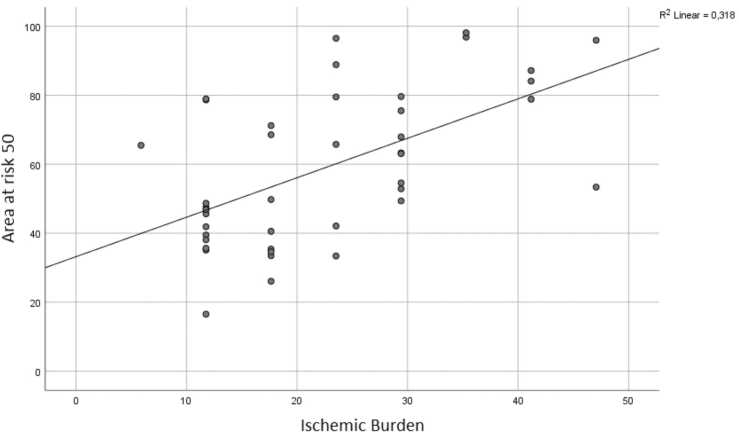
“Area at risk 50″ represents the percentage of myocardial area at risk of the total LV as calculated by using the Voronoi-based segmentation algorithm for every ≥ 50% stenosis. “Ischemic burden” represents the percentage of segments with relative hypoperfusion of the total amount of segments (=17).

**Fig. 6:**
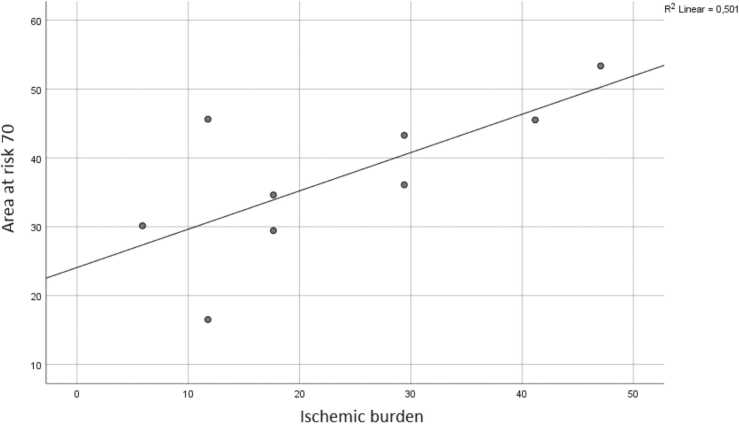
“Area at risk 70″ represents the percentage of myocardial area at risk of the total LV as calculated by using the Voronoi-based segmentation algorithm for every ≥ 70% stenosis. “Ischemic burden” represents the percentage of segments with relative hypoperfusion of the total number of segments (=17).

**Fig. 7:**
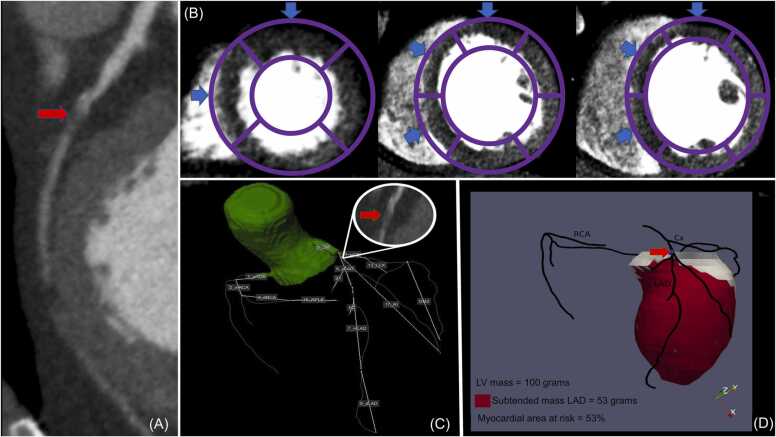
Example of a 58-year-old male with single vessel disease. A significant stenosis is present in the proximal LAD with contrast opacification distally (Panel A). Perfusion defects assessed by CTP can be seen in panel B. The ischemic burden can consequently be calculated as 8/17 * 100 ≈ 47%. The complete coronary tree with the relevant stenosis is shown in panel C. Using the previously mentioned stenosis the subtended mass is calculated by using the Voronoi-based segmentation algorithm. Subsequently, the myocardial area at risk is calculated as 53/100 * 100 = 53%.

CCTA and CTP images from forty-two patients (25 men, mean age, 68.2 ± 7.7) were used for the current analysis. Patient characteristics are listed in Table 1. Voronoi-based segmentation and semi-automatic drawing of the LV epi- and endocardial contours using a machine learning algorithm was successful in all cases. A total of 77 coronary lesions with a luminal stenosis of ≥ 50% were assessed. Average myocardial area at risk for stenosis ≥ 50% and ≥ 70% were 59% and 37%, respectively. Average ischemic burden for stenosis ≥ 50% and ≥ 70% were 23% and 24%, respectively. There was a moderate correlation of the ischemic burden versus myocardial area at risk for stenosis of ≥ 50% (r = 0.564; p < 0.01) (Fig. 5). A good correlation was found for the ischemic burden versus the area at risk for stenosis of ≥ 70% (r = 0.708; p < 0.01) (Fig. 6). A complete example is depicted in Fig. 7.

**Table 1:** CAD: Coronary artery disease. 1: Defined as luminal diameter stenosis of ≥ 50% on CCTA in one major epicardial coronary vessel. 2: Defined as luminal diameter stenosis of ≥ 50% on CCTA in two major epicardial coronary vessels. 3: Defined as luminal diameter stenosis of ≥ 50% on CCTA in three major epicardial coronary vessels.

Patient characteristics	N = 42
Male/Female	25 (60%) / 17 (40%)
Age (years)	68.2 ± 7.7
Hypertension	23 (55%)
Hyperlipidaemia	22 (52%)
Diabetes mellitus	9 (21%)
Family history of CAD	22 (52%)
Smoking	3 (7%)
Single-vessel disease^1^	24 (57%)
Double-vessel disease^2^	10 (24%)
Triple-vessel disease^3^	8 (19%)

Comparison of the LV mass as calculated from manually drawn contours versus contours drawn with the machine learning model demonstrated a very good correlation (r = 0.870; p < 0.01).

## 4 Discussion

This study assessed the relationship between myocardial area at risk at CCTA and ischemic burden as assessed at CTP. Our results demonstrate that calculating subtended mass using a Voronoi-based segmentation algorithm in combination with a machine learning algorithm for semi-automatically drawing LV epi- and endocardial contours at CCTA is feasible and its correlation to the ischemic burden as measured using a standard 17-segment model at CTP increases with increasing stenosis severity. Consequently, coronary CTA can be used not only to assess the degree of a coronary stenosis, but also for quantification of the subtended myocardial mass which may predict the ischemic burden without the need for a stress test. It should however be noted that the use of integrated diagnostics of CCTA and CTP is still better than CCTA alone as the first allows for both assessment of coronary stenosis as well as the presence of (reversible) ischemia. This is of great importance as not every coronary stenosis is hemodynamically significant [15].

Multiple studies have demonstrated that adding CTP to regular CCTA improves the detection of hemodynamically significant coronary lesions [16], [17]. For instance, Pontone et al. demonstrated that addition of CTP to CCTA improved the detection of functional significant coronary lesions. In a vessel-based model addition of CTP to CCTA yielded an improvement of specificity (94%; p < 0.001), positive predictive value (86%; p < 0.001), and accuracy (93%; p = 0.002). Similarly, in a patient-based model, improvements in specificity (83%; p < 0.001), positive predictive value (86%; p = 0.02), and accuracy (91%; p = 0.004) were also observed when stress CTP was combined with CCTA [16].

Aside from the degree of coronary stenosis there have been several studies assessing the relationship between the anatomical location of a coronary stenosis and the presence of myocardial ischemia. For instance, in a study by Tanabe et al. the combined diagnostic performance of coronary artery stenosis-subtended myocardial volume and myocardial blood flow (MBF) on CTP for detecting obstructive coronary artery disease was assessed. It was found that the AUC of the combined use of the subtended CTP myocardial blood flow and subtended mass was significantly higher than that of myocardial blood flow alone in the detection of hemodynamically significant stenoses (0.89 vs. 0.75, 0.77; p < 0.05) [18].

Ide et al. demonstrated the feasibility and validity of Voronoi-based tissue segmentation. It was found that CCTA based subtended myocardial mass calculated using a Voronoi-based segmentation algorithm closely corresponded to actual subtended mass measured on ex-vivo-sine hearts (r = 0.92, p = 0.02 for the left anterior descending artery (LAD); r = 0.96, p = 0.009 for the circumflex artery (CX); r = 0.96, p = 0.009 for the right coronary artery (RCA)) [19].

Semi-automatic segmentation of the LV using a machine learning model for defining epi- and endocardial contours has been validated extensively. Several studies have reported high comparability to a manual segmentation of the LV versus a machine learning approach [20], [21], [22], [23]. It must also be noted that manually drawing epi- and endocardial contours is a time-intensive process of usually around 20–30 min[20]. Semi-Automatic LV segmentation can speed up this process significantly as we have noted an execution time of approximately 1 min and 20 s

Kurata et al. also assessed the relationship between calculated subtended mass at CCTA using a Voronoi-based segmentation algorithm and ischemic burden as assessed by single photon emission computed tomography (SPECT). A moderate correlation was found between the calculated subtended mass and ischemic burden (r = 0.531; p = 0.001) which is only slightly lower compared to our results (r = 0.564; p < 0.01) [24]. Also, Fukuyama et al. performed a similar study by assessing the relationship between calculated subtended mass at CCTA using a Voronoi-based segmentation algorithm and ischemic burden as assessed by magnetic resonance imaging (MRI). A slightly better correlation was found when correlating subtended mass to ischemic burden (r = 0.73; p < 0.001) [25]. This difference in correlation may be partially explained by the fact that cardiac MRI perfusion is still superior to cardiac CTP in the detection of (reversible) ischemia [26].

Interestingly, in our study lesions with a diameter stenosis of 70% or more demonstrated a better correlation between the myocardial area at risk and ischemic burden compared to lesions with a diameter stenosis of 50% (r = 0.708 and r = 0.564 respectively). A similar observation was found by Fukuyama et al. [25]. This difference in correlation may be attributed to the fact that lesions with a greater diameter stenosis may cause more (reversible) ischemia and hereby enlarge the ischemic burden. Van Rosendael et al. clearly demonstrated the relationship between quantitative CCTA lesion measurements and myocardial ischemia at CTP. It was confirmed that increasing stenosis percentage by quantitative CCTA is positively correlated to myocardial ischemia [15]. Furthermore, a recent study by Bax et al. demonstrated that lesions in left sided coronary arteries with a larger diameter stenosis were often localized more distally in the subsequent vessel. Thus, explaining the better correlation for lesions with a diameter stenosis of 70% or more as these accompany for a lower subtended mass [27].

### 4.1 Limitations

This study has several limitations which are inherent to its retrospective design. Firstly, the amount of analyzed patients is small which may have influenced the strength of the statistical analysis. Hence, future studies with a larger number of patients will be required to clarify the significance of these findings in clinical practice. Selection bias may have been introduced as we only selected patients with reversible ischemia as diagnosed on CTP. Secondly, the subtended mass was calculated using the anatomical location of the relevant coronary lesion. This was independent of whether the lesion was hemodynamically significant or not. In case of multivessel disease the correlation between subtended mass and ischemic burden may have been biased as we solely selected the most proximal lesions for calculating the subtended mass. Of course, the most proximal lesions also encompass the largest subtended mass. Also, there was no validation of the ischemic burden to the corresponding anatomical territory that corresponds to the relevant coronary artery lesion used for calculating the myocardial area at risk [28]. Thirdly, the Voronoi-based segmentation algorithm does not take into account the curved surface of the myocardium but derives the distance the between the coronary vessels and every myocardial voxel by using a straight line. As distances are relatively small we feel the impact of not using the myocardial curvature on the final output will be very minimal. Lastly, we must acknowledge that no inter- or intra-observer measurements were done on the CCTA or CTP analysis. However, prior studies have reported excellent and moderate inter- and intra-observer agreements for both imaging modalities. [6], [29].

## 5 Conclusions

Quantification of the myocardial area at risk calculated by using a Voronoi-based algorithm in combination with a machine learning based algorithm for LV segmentation at CCTA significantly correlates with the ischemic burden as assessed by the standard 17-segment model at CTP. This correlation improves with increasing stenosis degree. This relationship may be beneficial in risk assessment of patients with CAD and may aid in clinical-decision making.

## Ethical statement

All data were retrospectively analyzed. The local ethics committee of the Leiden University Medical Center approved this retrospective analysis of clinical data and the need for informed consent was waived.

## Funding statement

This research did not receive any specific grants from funding agencies in the public, commercial, or not-for-profit sectors.

## CRediT authorship contribution statement

**Finn van Driest:** Conceptualization, Validation, Formal analysis, Writing – original draft, Writing – review & editing, Visualization. **Coen Bijns:** Writing – original draft. **Rob van der Geest:** Methodology, Software, Writing – review & editing. **Alexander Broersen:** Software, Writing – review & editing. **Jouke Dijkstra:** Software**,** Writing – review & editing. **Wouter Jukema:** Writing – review & editing, Supervision. **Arthur Scholte:** Writing – review & editing, Supervision.

## Conflicts of interest statement

All authors have nothing to disclose.
